# Polymorphism of Transferrin Gene Impacts the Mediating Effects of Psychotic Symptoms on the Relationship between Oxidative Stress and Cognition in Patients with Chronic Schizophrenia

**DOI:** 10.3390/antiox11010125

**Published:** 2022-01-06

**Authors:** Pinhong Chen, Dongmei Wang, Meihong Xiu, Dachun Chen, Blake Lackey, Hanjing E. Wu, Jin Zhou, Xiangyang Zhang

**Affiliations:** 1 Beijing Institute of Basic Medical Sciences, Beijing 100850, China; chenpinhong07@126.com; 2 CAS Key Laboratory of Mental Health, Institute of Psychology, Beijing 100101, China; wangdm@psych.ac.cn; 3 Department of Psychology, University of Chinese Academy of Sciences, Beijing 100101, China; 4 Beijing HuiLongGuan Hospital, Peking University, Beijing 100096, China; xiumeihong97@163.com (M.X.); cdc1963@sina.com (D.C.); 5 Department of Psychiatry and Behavioral Sciences, University of Texas Health Science Center at Houston, Houston, TX 77030, USA; Blake.n.lackey@uth.tmc.edu (B.L.); Hanjing.Wu@uth.tmc.edu (H.E.W.)

**Keywords:** transferrin, polymorphism, oxidative stress, psychotic symptoms, cognitive function, schizophrenia

## Abstract

A series of studies indicated that iron distribution that partly derives from transferrin-bound iron in the peripheral nervous system in the brain may act in processes such as myelination and brain development. However, the relationship between schizophrenia, its psychotic symptoms, and the transferrin (TF) gene has not been systematically explored. Our study aimed to investigate how a particular polymorphism of the transferrin gene, rs3811655, affects the superoxide dismutase (SOD), malondialdehyde (MDA), psychotic symptoms, cognition, or the mediation model between antioxidant enzymes and cognition via symptoms. A total of 564 patients with chronic schizophrenia and 468 healthy control subjects were recruited. The psychotic symptoms and cognition were assessed by the Positive and Negative Syndrome Scale (PANSS) and the Repeatable Battery for the Assessment of Neuropsychological Status (RBANS), respectively. Furthermore, the serum SOD, MDA activity, and transferrin gene polymorphism were measured in patients. Our results demonstrated that patients with the G allele possessed more severe negative symptoms, worse cognitive performance with respect to attention, and higher serum Mn-SOD activity. Additionally, the rs3811655 polymorphism may act as a moderator in the association between Cu/Zn-SOD activity and cognition, as well as psychotic symptoms in patients suffering from schizophrenia. According to this study, the single nucleotide polymorphism (SNP) rs3811655 polymorphism may fail to contribute to the susceptibility of schizophrenia in an individual but is involved in the iron-induced oxidative stress disturbance and cognitive impairment in schizophrenia. This deepens our understanding of the critical role of iron-induced oxidative stress that might underlie the pathophysiology of schizophrenia.

## 1. Introduction

Schizophrenia patients with severe psychotic symptoms perform poorly in cognitive tasks and cognition, which, in turn, correlates with social and vocational impairment [1]. For example, negative and disorganized symptoms are related to poor performance in verbal IQ and concept attainment [1]. Meta-analysis of large cohort studies has proved that prefrontal thickness was significantly correlated with negative symptom severity in schizophrenia after controlling the influences of age, gender, site, and overall illness severity [2]. However, twin studies and molecular genetic methods have indicated that the link between the majority of the genetic variance in schizophrenia and cognitive performance was small and represents a “weak” association between cognitive impairment and psychosis [3]. As a non-specific indicator of brain malfunction, cognitive deterioration is associated with psychosis, but it is not a trait that can be justified by conventional physiological understanding. Therefore, it is important to take other disease pathophysiology factors into account.

Besides cognitive function, psychiatric symptoms are likewise related to oxidative balance in schizophrenia patients. Previous evidence illustrated that thioredoxin, a key redox-regulating protein of antioxidants, was positively associated with positive symptoms of first-episode schizophrenic patients [4]. Another study confirmed that the two biomarkers of antioxidative defense, Mn-SOD and total SOD activity, were positively correlated with either the positive and negative syndrome scale (PNASS) depressive factor or the PNASS general psychopathology subscale score in the never-treated, first-episode schizophrenia patients [5]. In terms of a meta-analysis pooling with a series of randomized controlled trials of antioxidant supplementation, ginkgo significantly reduced the positive symptoms, while N-acetyl cysteine (NAC) significantly decreased the negative symptoms in trials involving schizophrenia [6]. These findings suggested that oxidative stress may be related to the pathogenesis of schizophrenia in some way, and antioxidants have been used to treat and prevent psychotic symptoms in schizophrenic patients.

Over recent years, oxidative stress has been determined as a possible factor in the neuropathological mechanism of schizophrenia [7]. Meanwhile, it has been reported that abnormal biological alterations of oxidative stress parameters across different schizophrenia samples [8,9] have been found. Based on increasing evidence, the impaired oxidative status may be implicated in abnormal behavior and cognitive dysfunction in patients with schizophrenia [10]. For instance, one recent study has found that oxidative stress markers in plasma may interact with brain-derived neurotrophic factor (BDNF) to influence executive dysfunction in patients with chronic schizophrenia [11]. Another study showed that the first-episode psychosis patients with high nitrite status and low glutathione levels exhibited worse executive function [12]. Moreover, a meta-analysis also demonstrated that the markers of lipid peroxidation and circulating copper were elevated when the total antioxidant capacity was decreased in subjects with Alzheimer’s disease or mild cognitive impairment [13]. Furthermore, a preclinical study displayed that diet-induced oxidative stress did not impair cognitive function in normal mice but did affect maze performance in a transgenic mouse model of ApoE−/− mice, which suggested that oxidative stress may damage the cognitive function directly [14].

Transferrin is an iron transport glycoprotein derived from hepatocytes and oligodendrocytes, and it has been regarded as a reliable index of the long-term iron metabolism in the brain [15]. Iron homeostasis is a necessary feature of viable brain monoaminergic functions, and iron deficiency or overload is thought to be associated with neuropsychiatric disorders with dopamine abnormality [16]. Microarray analysis studies have indicated a max LOD (logarithm of the odds) score of 2.0–3.0 for schizophrenia loci and transferrin loci, and it could be considered as sufficient evidence for linkage [17]. Several lines of evidence from genetic association studies implicated that brain transferrin could be involved in the pathophysiology of schizophrenia among different cohorts, including the Chinese Han population [18,19,20]. Previous findings revealed that the serum levels of transferrin were decreased in schizophrenia patients compared with normal controls [21]. Additionally, cumulative studies have proved the association between the polymorphism of the transferrin gene and psychotic symptoms [16], the engagement of transferrin in brain microstructure [22], and iron-induced oxidative stress [23]. However, the relationship between the polymorphism of the transferrin gene and core schizophrenia phenotypes has not been fully elucidated.

In view of the role of the iron-induced oxidative stress in the pathophysiology of schizophrenia, whether the rs3811655 polymorphism in the intron 3 of the transferrin gene might influence psychotic symptoms, cognition, and oxidative stress in schizophrenia, was investigated. Moreover, it was hypothesized that psychotic symptoms may play an intermediary role in the relationship between oxidative stress and cognitive function, and the mediational relationship might be varied by the polymorphism of the transferrin gene. Thus, the current study attempted to investigate (1) the impacts of the rs3811655 polymorphism on psychotic and cognitive symptoms, as well as serum oxidative stress parameters in chronic patients with schizophrenia; (2) whether psychotic symptoms would be a mediating factor of oxidative stress on cognition; (3) whether rs3811655 polymorphism would modulate the mediation model amongoxidative stress, symptoms, and cognition.

## 2. Materials and Methods

### 2.1. Subjects

A total of 564 patients with chronic schizophrenia were recruited from Beijing Huilongguan Psychiatric hospital and HeBei Province Veterans Psychiatric Hospital. All the patients had to satisfy the following criteria: (a) a diagnosis that met the DSM-IV criteria for schizophrenia; (b) minimum illness duration of 5 years; (c) Han Chinese ethnicity at the age of 18–75 years; (d) long-term antipsychotic treatment for at least 12 months. The mean antipsychotic dose for schizophrenia in our study (in chlorpromazine equivalents) was 433.6 ± 366.6 mg/day, and the percentage of typical and atypical antipsychotics was 16.8% and 83.2%, respectively (12 patients missing medication information). A total of 468 healthy volunteer subjects who were Han Chinese and aged from 18 to 75 years were enlisted from the local community in Beijing. The control subjects’ current mental status and personal or family history of mental disorders were obtained by a research assistant; none of the control subjects presented to be potential individuals with an Axis I disorder.

The exclusion criteria for all the subjects are as follows: (a) history of a neurological disorder; (b) severe head trauma; (c) uncontrolled diabetes, cerebrovascular disease, or cardiovascular disease; (d) drug or alcohol abuse/dependence, excepting tobacco.

The research protocol was approved by the Institutional Review Board of Beijing Huilongguan hospital. Informed consent was obtained from all subjects or guardians.

### 2.2. Clinical Examination and Cognitive Assessments

The Structured Clinical Interview for DSM-IV (SCID-I/P) was employed to screen each participant by two trained research assistants. A data collection protocol was used to gather demographics and clinical information. Researchers estimated psychotic symptoms of patients by using the Positive and Negative Syndrome Scale (PANSS) [24]. Cognitive performance was assessed by the translated Chinese version of the Repeatable Battery for the Assessment of Neuropsychological Status (RBANS) [25]. The RBANS consists of a total scale and five domain-specific scores for immediate memory, visuospatial/constructional, language, attention, and delayed memory. 

Before this study, four experienced psychiatrists received a training course using PANSS and RBANS. After training, their inter-rater correlation coefficient (ICC) on the PANSS or RBANS total score was 0.84 and 0.85, respectively.

### 2.3. Measurement of Antioxidant Enzymes Activity

About 5 mL of venous blood samples from 275 patients were collected for SOD activity testing. SOD activity and MDA levels were detected by the T-SOD assay kit and the MDA assay kit (Nanjing Jiangcheng Bioengineering Institute, Nanjing, China). All samples were labeled with a unique code number instead of personal information and were assayed by the same technician who was blinded to the conditions.

### 2.4. DNA Extraction and SNP Detection

The genomic DNA was extracted from peripheral blood leukocyte pellets of 564 schizophrenia and 422 controls (with 46 cases missing) using the salting-out strategy [26] and later stored at −80 degrees. By following the method in our former study, the transferrin gene polymorphism rs3811655 was genotyped utilizing Matrix-Assisted Laser Desorption/Ionization Time of Flight Mass Spectrometry (MALDI-TOF MS) in the MassARRAY System (Sequenom Inc., San Diego, CA, USA). To monitor and evaluate the DNA sequencing procedure, repeated genotyping of randomly selected 5% of samples was carried out, and the discordance rate was 0.1%.

### 2.5. Statistical Analysis

The between-group differences in demographics were checked using one-way ANOVA for continuous variables and chi-square for categorical variables. Pearson’s chi-square (χ^2^) test was used to assess whether patients and control groups in our study followed the Hardy–Weinberg equilibrium (HWE). The between-group differences in genotype and allele frequencies were tested using the χ^2^ tests. Additionally, a logistic regression analysis was conducted to assess whether the distribution of the rs3811655 genotype was significantly different between groups after controlling age, gender, education, and smoking.

Multivariate analysis of variance (MANOVA) was undertaken with psychotic symptoms/cognition/oxidative stress as the independent variables and genotype as the dependent variables and controlled for confounders, including gender, age, education, the status of smoking, and pharmacological treatment. Bonferroni correction was applied for multiple tests. Partial correlation analysis was also performed to identify the relationship among psychotic symptoms, cognition, and oxidative stress.

Lastly, a series of stepwise logistic regressions were performed to determine whether psychotic symptoms could mediate the relationship between oxidative stress and cognitive performance among schizophrenia patients. The mediation effect measurements were then carried out separately for C allele carriers and carriers with at least one copy of the G allele for the rs3811655 SNP. All analyses of mediation were adjusted for demographic and pharmacological variables that may exert a potential impact on cognition.

## 3. Results

### 3.1. Subject Characteristics and Genotypic Effects on Susceptibility to Schizophrenia

The demographic characteristics and genotype distributions between cases and controls are illustrated in Table 1. No significant difference was found in body mass index data, but the two groups were distributed unequally with respect to gender, age, education, and smoking status (all *p* < 0.05). These variables, plus pharmacological treatment variables (types and chlorpromazine equivalent doses of antipsychotics), were adjusted to avoid potential confounding biases in the subsequent analyses. The χ^2^ goodness of fit test showed that both allele and genotype frequencies of the rs3811655 polymorphism were in Hardy–Weinberg equilibrium in patients and controls (both *p* > 0.05). After adjusting for confounding factors, there were no significant differences in the genotype distribution or allele frequency between the two groups (both *p* > 0.05).

### 3.2. TF Gene Polymorphisms and Clinical Symptoms of Schizophrenia

As shown in Table 2, no significant genotypic differences in the PANSS total and its subscale scores were noted except for the N subscore (*F* = 4.37, *p* < 0.05). The rs3811655 genotype accounted for 1.6% of the variance (adjusted *R*^2^ = 0.016). The carriers of at least one copy of the G allele exhibited more severe negative psychopathology than that of the homozygous for the C allele (both *p* < 0.05). There are significant genotypic effects on the RBANS attention subscore (*F* = 6.18, *p* < 0.05) among schizophrenia (Bonferroni corrected all *p* < 0.05). The rs3811655 genotype accounted for 2.2% of the variance (adjusted *R*^2^ = 0.022). The GG genotype group had a lower attention index score than the CC and GC genotype groups in these patients (both *p* < 0.01). Furthermore, the mean SOD and MDA levels were available from 275 patients. MANOVA analysis showed the significant genotypic effects on Mn-SOD activity (*F* = 4.95, *p* < 0.01), and the rs3811655 genotype accounted for 3.6% of the variance in patients (adjusted *R*^2^ = 0.036). The GG genotype carriers displayed a higher Mn-SOD level than the CC and GC genotypes (both *p* < 0.05).

### 3.3. Correlation among Psychotic Symptoms, Cognitive Performance, and Oxidative Stress in Schizophrenia Patients

Partial correlation showed that the PANSS total score was positively associated with Cu/Zn-SOD activity (*r* = 0.16, *p* < 0.01) and negatively correlated with the serum level of Mn-SOD (*r* = −0.12, *p* < 0.05), and there was an obvious negative association between the PANSS total score and RBANS total score (*r* = −0.23, *p* < 0.001) in patients with chronic schizophrenia (Table 3).

### 3.4. Mediation Effects of Psychotic Symptoms on the Relationship between Oxidative Stress and Cognitive Performance

**Figure 1:**
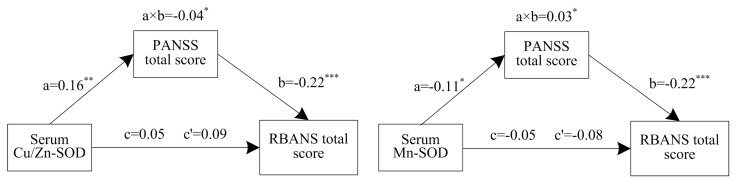
Mediation models: The relationship between serum Cu/Zn–SOD/ Mn–SOD and cognition through psychotic symptoms. a × b: the indirect effects of oxidative stress on cognition; c = the total effects of oxidative stress on cognition; c’ = the direct effects of oxidative stress on cognition; a = the effects of oxidative stress on psychotic symptoms; b = the effects of psychotic symptoms on cognition. * *p* < 0.05, ** *p* < 0.01, *** *p* < 0.001.

As indicated by the Baron and Kenny method, a set of stepwise logistic regressions were then carried out in all patients with schizophrenia for the mediation analysis to verify whether the psychotic symptoms could mediate the relationship between oxidative stress and cognitive performance (shown in Figure 1).There were significant indirect effects of Cu/Zn-SOD activity via psychotic symptoms on the RANSS total score (a × b = −0.04), and bootstrapping (resamples = 5000) analysis revealed that the bootstrap confidence did not include zero (95% CI [−0.08, −0.01]), which indicated significant indirect effects. There were significant direct effects of serum Cu/Zn-SOD on psychotic symptoms (a = 0.16, *p* < 0.01) and great direct effects of psychotic symptoms on the RANSS total score (b = −0.22, *p* < 0.001). Additionally, Cu/Zn-SOD did not significantly predict cognitive performance (c = 0.05, *p* > 0.05), and after controlling for the effect of psychotic symptoms, the relationship between oxidative stress and cognition remained nonsignificant (c’ = 0.09, *p* > 0.05), indicating a complete mediation effect.

Furthermore, the serum Mn-SOD exhibited significant indirect effects on the RANSS total score through psychotic symptoms (a × b = 0.03, 95% CI [0.005,0.066]), and the serum Mn-SOD also exerted a direct effect on psychotic symptoms (a = −0.11, *p* < 0.05). Moreover, psychotic symptoms fully mediated the link between the serum Mn-SOD activity and RBANS total score (without a mediator: c = −0.05, *p* > 0.05; with a mediator: c’ = −0.08, *p* > 0.05).

### 3.5. The Mediating Effects Varied with Transferrin rs3811655 Polymorphism

**Figure 2:**
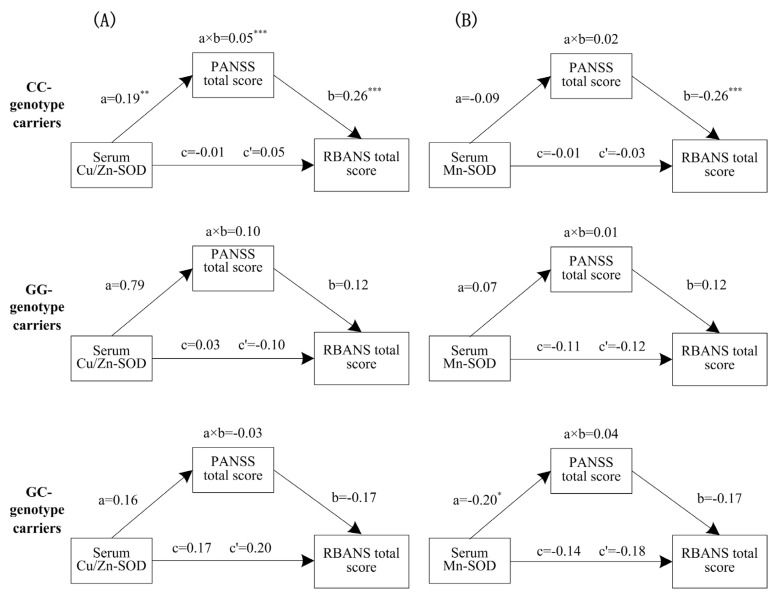
Mediation path analysis among serum Cu/Zn–SOD or Mn–SOD, psychotic symptoms and cognition varied with transferrin rs3811655 polymorphism. (**A**): Mediation model of serum Cu/Zn–SOD via psychotic symptoms on cognition. (**B**): Mediation model of serum Mn–SOD via psychotic symptoms on cognition. a × b: the indirect effects of oxidative stress on cognition; c = the total effects of oxidative stress on cognition; c’ = the direct effects of oxidative stress on cognition; a = the effects of oxidative stress on psychotic symptoms; b = the effects of psychotic symptoms on cognition. * *p* < 0.05, ** *p* < 0.01, *** *p* < 0.001.

In addition to assessing the mediation role of psychotic symptoms on the relations between serum Cu/Zn-SOD/ Mn-SOD and cognition, we examined whether these mediation models would act differently with the rs3811655 genotype.For the C allele carriers, serum Cu/Zn-SOD exerted a significant indirect effect on the RANSS total score via psychotic symptoms (a × b = 0.05, 95% CI [−0.13, −0.01]) (Figure 2A). There were significant direct effects of Cu/Zn-SOD activity on psychotic symptoms (a = 0.19, *p* < 0.01) and psychotic symptoms on the RANSS total score (b = 0.26, *p* < 0.001), and no significant direct effects of Cu/Zn-SOD activity on cognition (c’ = 0.05, *p* > 0.05). Overall, these results indicated that among C allele carriers from the schizophrenia group, psychotic symptoms totally mediated the impacts of Cu/Zn-SOD activity on cognitive function.

For respondents with the rs3811655-GG/GC genotypes, there were no significant indirect effects of serum Cu/Zn-SOD on cognition via symptoms, no significant effects of Cu/Zn-SOD on symptoms and symptoms on cognition, and no significant direct effects of Cu/Zn-SOD on cognition.

Moreover, the hypothesis of the influences of transferrin rs3811655 polymorphism on the mediation model of serum Mn-SOD on schizophrenia patients’ cognition via psychotic symptoms was investigated (Figure 2B). The analysis indicated that regardless of whether respondents carried the transferrin rs3811655 C allele or were carriers of at least one copy of the G allele, there were no mediating effects of psychotic symptoms on the links between oxidative stress and cognition.

## 4. Discussion

In this study, a mediation model was used to examine the iron-induced oxidative stress mechanisms by which antioxidant enzymes and rs3811655 genotype interact to alter schizophrenic symptoms or cognition. It was demonstrated that (a) there was no significant correlation between transferrin gene polymorphism and the susceptibility to schizophrenia; (b) rs3811655-G variant was associated with more severe symptoms, worse cognition, and higher SOD activity in schizophrenia; (c) mediation analyses indicated that psychotic symptoms played a fully intermediary role between either Cu/Zn-SOD or Mn-SOD activity and cognition; (d) the mediating role of psychotic symptoms on the relations between Cu/Zn-SOD and cognition may vary with the transferrin gene polymorphism (rs3811655).

### 4.1. More Severe Symptoms, Worse Cognition, and Higher SOD Activity in Schizophrenia Patients Are Associated with the rs3811655-G Variant

In this present study, it was also observed that the G allele carriers of the variant rs3811655 were associated with more severe negative symptoms, worse cognitive performance with respect to attention, and higher serum Mn-SOD activity in schizophrenia. Previous studies have shown that serum transferrin levels are closely correlated with the changes in the local brain volume and white matter integrity of healthy young people, which indicated that iron homeostasis plays a key role in the developing brain [27]. Other works have reported that genes related to iron metabolism, such as the TF and hemochromatosis (HFE) genes, may explain nearly 40% of the genetic variation in serum transferrin levels [28]. The GG variant of the transferrin gene polymorphism is associated with lower serum transferrin levels [29]. Therefore, as a principal transport pathway for iron, the transferrin gene may be related to altered serum transferrin production and increased hydroxyl radicals, which might attack nucleic acids, proteins, or lipid species, thus causing tissue damage, known as oxidative stress disturbance [13,30]. Superoxide dismutase activity was found to be significantly lower in drug-naïve, first-episode schizophrenia patients but higher in chronic patients [31,32]. Additionally, meta-analysis has shown convincing evidence of elevated oxidative stress in serum, erythrocytes, and circulating lymphocytes in Alzheimer’s disease and mild cognitive impairment [13]. Similarly, another meta-analysis of a large sample demonstrated that patients with Parkinson’s disease exhibited significantly increased blood concentrations of biomarkers of oxidative stress and iron metabolism, including 8-hydroxy-2’ -deoxyguanosine (8-OHdG), malondialdehyde (MDA), nitrite, and ferritin [33]. Moreover, iron-induced oxidative impairment may disrupt essential pathways of brain diseases, involving inflammatory responses, mitochondrial and oligodendrocyte dysfunctions, epigenetic changes, the overactivation of N-methyl-D-aspartate (NMDA) glutamate receptors, and the deterioration of fast-spiking gamma-aminobutyric acid (GABA) interneurons. All these processes are potentially important in promoting susceptibility to schizophrenia and its behavior phenotypes [21]. However, the explicit relationship among the rs3811655 polymorphism, SOD, psychotic symptoms, and cognition deserve to be further discussed.

### 4.2. The Mediating Role of Psychotic Symptoms between SOD and Cognition

In the current study, the indirect effects showed that Cu/Zn-SOD or MnSOD activity predicted cognition negatively or positively via psychotic symptoms while the effects of serum Cu/Zn-SOD or MnSOD on cognition were completely mediated by psychotic symptoms. Additionally, we also found that impaired cognition is associated with increased psychotic symptoms, and the PANSS total score correlated positively with serum Cu/Zn-SOD and negatively with serum Mn-SOD. Accordingly, other groups have also exhibited a significant correlation between Cu/Zn-SOD or Mn-SOD and psychotic symptoms but a correlation in opposite directions [5,34]. The reasons for this controversy may be due to disease states, the use of different antipsychotic drug types (typical or atypical) [35], or the heterogeneity of psychiatric symptom evaluation tools. Cu/Zn-SOD and Mn-SOD are, respectively, located in the mitochondrial inner membrane space and the mitochondrial matrix, and they serve as enzymes catalyzing the dismutation of the superoxide radical (O_2_^●−^) into ordinary molecular oxygen (O_2_) and hydrogen peroxide (H_2_O_2_) [36]. The abnormal increase in intracellular reactive oxygen species causes a compensation response of the antioxidant defense system, localized inflammation, or cytokine release, as well as mtDNA release from the mitochondria, which is a result of mitochondrial dysfunction or impaired mitophagy [37]. For example, a study employing parallel transcriptomics, proteomics, and metabolomics approaches with fresh-frozen prefrontal cortex tissue has confirmed that genes related to energy metabolism and oxidative stress could differentiate almost 90% of schizophrenia patients from controls. Consequently, oxidative stress and mitochondrial dysfunction may result in global oxygen or glucose metabolic disturbances within the prefrontal cortex and may underlie the cognitive decline in schizophrenia [38]. Taken together, the aberrance of antioxidant systems may indicate unbalanced oxygen or glucose supply, which might be linked to the clinical syndrome of schizophrenia, thus contributing to poorer cognitive performance.

### 4.3. The Mediation Model of Psychotic Symptoms Moderated by Transferrin Gene Polymorphism

Additionally, this study confirmed that the rs3811655 polymorphism moderated the association between Cu/Zn-SOD, rather than Mn-SOD activity, and cognition through psychotic symptoms in schizophrenia. Moreover, the mediation model of Cu/Zn-SOD on the cognitive performance was proved among those with CC genotype but was absent in GG or GC carriers. A case-control association analysis suggested that statistically significant differences in both allele and genotype frequencies were found in SNP rs3811655, and the frequency of allele C was much higher than that of allele G [19]. Patients’ frequency of allele C or G in this study were 78.5% and 21.5%, respectively, thus leading to the conclusion that, perhaps, it is the larger representation of the C allele that makes the mediated relationship between Cu/Zn-SOD and cognition more significant and prominent. Specifically, these moderated mediation effects in Cu/Zn-SOD by variants in the transferrin gene were only found, which suggested that the carriers of the CC allele were more sensitive to the iron-induced oxidative stress processes in which Cu/Zn-SOD may play an important role in the development of psychosis and cognitive dysfunction. Therefore, in the presence of CC genotype, patients with high Cu/Zn-SOD activity might experience more severe psychotic symptoms and decline of social function and, thus, may exhibit more cognitive dysfunction.

The limitations of the present study should be addressed: (1) a relatively small sample size was included in the present study, so the association between SNP and schizophrenia-related phenotypes may be exploratory, further analyses with larger sample sizes may characterize this relationship more definitively; (2) female patients accounted for a relatively small proportion of the overall patients, which may lead to insufficient representative samples; (3) psychotic symptoms and cognitive impairment of schizophrenia were confirmed to be associated with medication, metabolic markers [39], and co-existing diseases like thyroid diseases [40]: although pharmacological treatment variables were included as covariates in statistics, the effects of the above variables may affect the schizophrenia symptoms, cognitive phenotypes, and their interrelationships; (4) the cross-sectional design of this study cannot confirm the causality or mediating effects, longitudinal design research will help clarify causality; (5) as this study only took the effect of a single SNP site into account, we must consider that the development of schizophrenia may be related to multiple genes and environmental factors, which need further research.

## 5. Conclusions

In summary, our results indicated that transferrin gene polymorphism facilitated superoxide dismutase activity, psychotic symptoms, and cognitive performance. Despite this, transferrin gene polymorphism moderated the association between Cu/Zn-SOD activity and cognition via psychotic symptoms in Chinese patients with chronic schizophrenia. As far as we know, this is the first study to systematically explore the influences of transferrin gene polymorphism on the antioxidant system, symptoms, and cognition, as well as the mechanism of their relationship in patients with schizophrenia.

## Figures and Tables

**Table 1:** Demographics and genotype distributions of the participants.

Variable	Schizophrenia (n = 564)	Healthy Control (n = 468)	t/χ^2^	*p*
Gender (M/F)	499/65	194/274	256.38	0.00 ***
Age (years)	47.8 ± 9.0	44.9 ± 13.6	−3.97	0.00 ***
Education (years)	8.9 ± 4.5	9.7 ± 5.3	2.40	0.02 **
Body mass index (kg/m^2^)	24.8 ± 3.9	25.1 ± 4.1	1.14	0.26
Smokers (%)	396/564	161/468	132.04	0.00 ***
Mean antipsychotic dose (as CPZ equivalents) (mg/d)	433.6 ± 366.6			
Types of antipsychotics (typical/atypical)	93/459			
TF allele frequency (%)				
C	78.5%	78.2%	0.03	0.85
G	21.5%	21.8%		
TF genotype distribution [n (%)]				
CC	350 (62.0%)	256 (60.6%)	0.64	0.73
GG	28 (5.0%)	18 (4.3%)		
GC	186 (33.0%)	148 (35.1%)		

** *p* < 0.01, *** *p* < 0.001.

**Table 2:** Genotypic effects on psychotic symptoms, cognitive functions, and biological markers of oxidative stress in patients.

5	Schizophrenia			
	C/C (n = 350)	G/G (n = 28)	G/C (n = 186)	F/χ^2^ (*p*)
PANSS scores				
P subscore	11.1 ± 4.6	11.0 ± 4.9	11.4 ± 5.0	0.47 (0.62)
N subscore	21.7 ± 7.2	25.4 ± 7.3	23.0 ± 7.3	4.37 (<0.05)
G subscore	24.6 ± 5.2	24.7 ± 4.8	25.2 ± 6.0	0.89 (0.41)
total score	57.3 ± 13.0	61.2 ± 11.7	59.5 ± 14.7	2.42 (0.09)
RBANS Scores				
Immediate Memory	58.6 ± 16.2	54.8 ± 13.9	59.2 ± 16.5	0.51 (0.60)
Visuospatial/Constructional	77.6 ± 18.8	73.1 ± 20.8	77.7 ± 18.2	0.95 (0.39)
Language	81.7 ± 15.8	76.8 ± 15.0	82.1 ± 14.4	1.42 (0.24)
Attention	71.4 ± 17.5	61.4 ± 14.7	70.3 ± 17.4	6.18 (<0.001)
Delayed Memory	67.2 ± 19.0	63.5 ± 19.1	65.6 ± 19.8	0.85 (0.43)
Total scale	64.7 ± 15.1	60.4 ± 12.7	64.5 ± 14.9	1.33 (0.27)
Biological Markers of Oxidative Stress	C/C (n = 168)	G/G (n = 11)	G/C (n = 96)	
Total SOD activity	77.4 ± 8.3	80.2 ± 14.6	77.8 ± 11.6	0.37 (0.69)
Cu/Zn-SOD activity	57.4 ± 17.1	45.4 ± 24.9	53.9 ± 19.6	2.35 (0.10)
Mn-SOD activity	20.0 ± 14.7	34.8 ± 18.0	23.9 ± 16.0	4.95 (<0.05)
MDA content	2.3 ± 2.0	3.0 ± 1.9	2.1 ± 8.1	0.23 (0.79)

SOD, superoxide dismutase; MDA, Malondialdehyde.

**Table 3:** Partial correlation among psychotic symptoms cognitive performance and oxidative stress in schizophrenia patients.

	PANSS Total Score	RBANS Total Score	Total SOD	Cu/Zn-SOD	Mn-SOD	MDA Content
PANSS total score	-					
RBANS total score	−0.23 ***	-				
Total SOD	0.12	0.01	-			
Cu/Zn-SOD	0.16 **	0.05	0.54 ***	-		
Mn-SOD	−0.12 *	−0.06	−0.01	−0.85 ***	-	
MDA content	−0.04	−0.09	−0.05	−0.07	0.05	-

* *p* < 0.05, ** *p* < 0.01, *** *p* < 0.001.

## Data Availability

Data is contained within the article.

## References

[1] O’Leary D.S., Flaum M., Kesler M.L., Flashman L.A., Arndt S., Andreasen N.C. (2000). Cognitive correlates of the negative, disorganized, and psychotic symptom dimensions of schizophrenia. J. Neuropsychiatry Clin. Neurosci..

[2] Walton E., Hibar D.P., van Erp T.G., Potkin S.G., Roiz-Santiañez R., Crespo-Facorro B., Suarez-Pinilla P., Van Haren N.E.M., de Zwarte S.M.C., Kahn R.S. (2018). Left medial orbitofrontal cortical thinning is associated with negative symptom severity in schizophrenia: A meta-analysis by the ENIGMA-Schizophrenia consortium. Psychol. Med..

[3] Reichenberg A., Velthorst E., Davidson M. (2019). Cognitive impairment and psychosis in schizophrenia: Independent or linked conditions?. World Psychiatry.

[4] Zhang X.Y., Xiu M.H., Wang F., Chen D.C., Qi L.Y., Sun H.Q., Chen S., He S.C., Wu G.Y., Haile C.N. (2009). The novel oxidative stress marker thioredoxin is increased in first-episode schizophrenic patients. Schizophr. Res..

[5] Lang X., Wang D.M., Du X.D., Fang Q.J., Chen D.C., Xiu M., Wang L., Zhang X.Y. (2020). Elevated activity of plasma superoxide dismutase in never-treated first-episode schizophrenia patients: Associated with depressive symptoms. Schizophr. Res..

[6] Magalhães P.V., Dean O., Andreazza A.C., Berk M., Kapczinski F. (2016). Antioxidant treatments for schizophrenia. Cochrane Database Syst. Rev..

[7] Maas D., Vallès A., Martens G. (2017). Oxidative stress, prefrontal cortex hypomyelination and cognitive symptoms in schizophrenia. Transl. Psychiatry.

[8] Flatow J., Buckley P., Miller B.J. (2013). Meta-analysis of oxidative stress in schizophrenia. Biol. Psychiatry.

[9] Zhang M., Zhao Z., He L., Wan C. (2010). A meta-analysis of oxidative stress markers in schizophrenia. Sci. China Life Sci..

[10] Murray A.J., Rogers J.C., Katshu M.Z.U.H., Liddle P.F., Upthegrove R. (2021). Oxidative stress and the pathophysiology and symptom profile of Schizophrenia Spectrum Disorders. Front. Psychiatry.

[11] Wei C., Sun Y., Chen N., Chen S., Xiu M.H., Zhang X.Y. (2020). Interaction of oxidative stress and BDNF on executive dysfunction in patients with chronic schizophrenia. Psychoneuroendocrinology.

[12] Martínez-Cengotitabengoa M., Mac-Dowell K.S., Leza J.C., Micó J.A., Fernandez M., Echevarria E., Sanjuan J., Elorza J., González-Pinto A. (2012). Cognitive impairment is related to oxidative stress and chemokine levels in first psychotic episodes. Schizophr. Res..

[13] Schrag M., Mueller C., Zabel M., Crofton A., Kitsch W.M., Ghribi O., Squitti R., Perry G. (2013). Oxidative stress in blood in Alzheimer’s disease and mild cognitive impairment: A meta-analysis. Neurobiol. Dis..

[14] Evola M., Hall A., Wall T., Young A., Grammas P. (2010). Oxidative stress impairs learning and memory in apoE knockout mice. Pharmacol. Biochem. Behav..

[15] Ahluwalia N. (1998). Diagnostic utility of serum transferrin receptors measurement in assessing iron status. Nutr. Rev..

[16] Kim S.-W., Stewart R., Park W.-Y., Jhon M., Lee J.-Y., Kim S.-Y., Kim J.-M., Amminger P., Chung Y.-C., Yoon J.-S. (2018). Latent iron deficiency as a marker of negative symptoms in patients with first-episode schizophrenia spectrum disorder. Nutrients.

[17] Davis K.L., Stewart D.G., Friedman J.I., Buchsbaum M., Harvey P.D., Hof P.R., Buxbaum J., Har4outunian V. (2003). White matter changes in schizophrenia: Evidence for myelin-related dysfunction. Arch. Gen. Psychiatry.

[18] Maeno N., Takahashi N., Saito S., Ji X., Branko A., Ishihara R., Yoshida K., Inada T., Iidaka T., Ozaki N. (2007). Association study between the transferrin gene and schizophrenia in the Japanese population. Neuro Rep..

[19] Qu M., Yue W., Tang F., Wang L., Han Y., Zhang D. (2008). Polymorphisms of Transferrin gene are associated with schizophrenia in Chinese Han population. J. Psychiatr. Res..

[20] Buretić-Tomljanović A., Vraneković J., Rubeša G., Jonovska S., Tomljanovic D., Sendula-Jengic V., Kapovic M., Ristic S. (2012). HFE mutations and transferrin C1/C2 polymorphism among Croatian patients with schizophrenia and schizoaffective disorder. Mol. Biol. Rep..

[21] Bitanihirwe B.K., Woo T.-U.W. (2011). Oxidative stress in schizophrenia: An integrated approach. Neurosci. Biobehav. Rev..

[22] Jahanshad N., Kohannim O., Hibar D.P., Stein J.L., McMahon K.L., de Zubicaray G.I., Medland S.E., Montgomery G.W., Whitfield J.B., Martin N.G. (2012). Brain structure in healthy adults is related to serum transferrin and the H63D polymorphism in the HFE gene. Proc. Natl. Acad. Sci. USA.

[23] Yamaji Y., Nakazato Y., Oshima N., Hayashi M., Saruta T. (2004). Oxidative stress induced by iron released from transferrin in low pH peritoneal dialysis solution. Nephrol. Dial. Transplant..

[24] Kay S.R., Fiszbein A., Opler L.A. (1987). The positive and negative syndrome scale (PANSS) for schizophrenia. Schizophr. Bull..

[25] Randolph C., Tierney M.C., Mohr E., Chase T.N. (1998). The Repeatable Battery for the Assessment of Neuropsychological Status (RBANS): Preliminary clinical validity. J. Clin. Exp. Neuropsychol..

[26] Miller S.A., Dykes D., Polesky H. (1988). A simple salting out procedure for extracting DNA from human nucleated cells. Nucleic Acids Res..

[27] Jahanshad N., Rajagopalan P., Thompson P.M. (2013). Neuroimaging, nutrition, and iron-related genes. Cell. Mol. Life Sci..

[28] Benyamin B., McRae A.F., Zhu G., Gordon S., Henders A.K., Palotie A., Peltonen L., Martin N.G., Montgomery G.W., Whitfield J.B. (2009). Variants in TF and HFE explain ~40% of genetic variation in serum-transferrin levels. Am. J. Hum. Genet..

[29] Wysokinski D., Danisz K., Blasiak J., Dorecka M., Romaniuk D., Szaflik J., Szaflik J.P. (2013). An association of transferrin gene polymorphism and serum transferrin levels with age-related macular degeneration. Exp. Eye Res..

[30] Zeng L., Xia T., Hu W., Chen S., Chi S., Lei Y., Liu Z. (2018). Visualizing the regulation of hydroxyl radical level by superoxide dismutase via a specific molecular probe. Anal. Chem..

[31] Raffa M., Mechri A., Othman L.B., Fendri C., Gaha L., Kerkeni A. (2009). Decreased glutathione levels and antioxidant enzyme activities in untreated and treated schizophrenic patients. Prog. Neuro-Psychopharmacol. Biol. Psychiatry.

[32] Zhang X.Y., Zhou D.F., Cao L.Y., Zhang P.Y., Wu G.Y., Shen Y.C. (2003). The effect of risperidone treatment on superoxide dismutase in schizophrenia. J. Clin. Psychopharmacol..

[33] Popa-Wagner A., Mitran S., Sivanesan S., Chang E., Buga A.-M. (2013). ROS and brain diseases: The good, the bad, and the ugly. Oxid. Med. Cell. Longev..

[34] Xiu M.H., Li Z., Chen D.C., Chen S., Curbo M.E., Wu H.E., Tong Y.S., Tan S.P., Zhang X.Y. (2020). Interrelationships between BDNF, superoxide dismutase, and cognitive impairment in drug-naive first-episode patients with schizophrenia. Schizophr. Bull..

[35] Hendouei N., Farnia S., Mohseni F., Salehi A., Bagheri M., Shadfar F., Barzegar F., Hoseini S.D., Charati J.Y., Shaki F. (2018). Alterations in oxidative stress markers and its correlation with clinical findings in schizophrenic patients consuming perphenazine, clozapine and risperidone. Biomed. Pharmacother..

[36] Mondola P., Damiano S., Sasso A., Santillo M. (2016). The Cu, Zn superoxide dismutase: Not only a dismutase enzyme. Front. Physiol..

[37] Moya G.E., Rivera P.D., Dittenhafer-Reed K.E. (2021). Evidence for the role of mitochondrial DNA release in the inflammatory response in neurological disorders. Int. J. Mol. Sci..

[38] Prabakaran S., Swatton J., Ryan M., Huffaker S.J., Huang J.T.-J., Griffin J.L., Wayland M., Freeman T., Dudbridge F., Lilley K.S. (2004). Mitochondrial dysfunction in schizophrenia: Evidence for compromised brain metabolism and oxidative stress. Mol. Psychiatry.

[39] Trześniowska-Drukała B., Kalinowska S., Safranow K., Kloda K., Misiak B., Samochowiec J. (2019). Evaluation of hyperhomocysteinemia prevalence and its influence on the selected cognitive functions in patients with schizophrenia. Prog. Neuro-Psychopharmacol. Biol. Psychiatry.

[40] Kalinowska S., Trześniowska-Drukała B., Safranow K., Pełka-Wysiecka J., Kloda K., Misiak B., Samochowiec J. (2019). Association between thyroid function and metabolic syndrome in male and female schizophrenia patients. Psychiatry Res..

